# Optimisation in fluoroscopy

**DOI:** 10.2349/biij.3.2.e47

**Published:** 2007-04-01

**Authors:** B Axelsson

**Affiliations:** Department of Medical Physics and Biomedical Engineering, Central Hospital, Växjö, Sweden

**Keywords:** Radiation protection, fluoroscopy, patient dose, dose reduction

## Abstract

Optimisation of radiation protection in fluoroscopy is important since the procedure could lead to relatively high absorbed doses both in patients and personnel resulting in acute radiation injury. Optimisation procedures include adjustment of the fluoroscopy equipment such as exposure factors as well as proper use of automatic brightness control and pulsed fluoroscopy. It is also important to gain the benefits of image processing and the higher sensitivity of flat panel detectors as compared to image intensifier-TV systems.

Proper positioning of the patient with respect to detector and X-ray tube is of fundamental importance to image quality and radiation dose to the patient. Both image quality and radiation dose are also affected by the methodology used with parameters such as magnification factor, increased filtration, use of last-image-hold and the use of a grid.

There is a direct relation between patient dose and the absorbed dose to the personnel since this is mostly due to scattered radiation from the patient. If the correct methodology and the correct radiation protection devices are used, the absorbed dose to the personnel could be minimised to acceptable levels even for those working with complex procedures.

In order to have an organised review of all aspects of optimisation, it is recommendable to have an active quality system at the department. This system should define responsibilities and tasks for persons involved.

## INTRODUCTION

Fluoroscopy is being used not only by radiologists but also by an increasing number of clinicians, for instance in interventional radiology. To obtain optimal benefit from the use of fluoroscopy without undue risk to the patient, it is important that the personnel have a thorough knowledge about the functioning and performance of the equipment and also adequate training in radiation protection and an awareness of the potential for injury both to the patient and personnel. It is widely known that there may be substantial differences in image quality and radiation dose among different institutions for the same type of procedure depending on the level of training and methodology [1, 2]. Some aspects on optimisation of image quality and radiation protection are discussed in this presentation.

## RADIATION DOSE AND BIOLOGICAL EFFECTS

Radiation exposure to the patient could be characterised by the dose-area product (DAP) or the entrance skin dose (ESD). DAP is the product of the absorbed dose in air by the area of the beam and is a measure of the total amount of radiation emitted from the equipment towards the patient. DAP could be used to calculate the effective dose, which characterises stochastic risk such as radiation-induced cancer. Cardiac catheterisation procedures, for instance, have been reported to deliver DAP of about 60 Gy/cm^2^, which would result in an effective dose of about 12 mSv [3]. ESD is used to evaluate the risk for deterministic effects such as skin lesions. ESD of 1-2.5 Gy has been reported for coronary interventions [4]. In recent years, there were also several reports on radiation-induced deterministic effects on patients as a result of complex interventional procedures [5, 6,]. For procedures where the ESD is estimated at or above 3 Gy (1Gy for repeated procedures), there should be a system to establish the maximum skin dose. These calculations should be indicated in the patients’ notes and the patients should also be reviewed between 10 and 14 days after the treatment.

Radiation exposure to the personnel is characterised by the absorbed dose to organs of interest such as hands or eye lens, or by the effective dose. High radiation doses to the hands and to the eye lens as well as deterministic effects have been reported with some procedures [7].

## EQUIPMENT FACTORS

Modern fluoroscopy equipment gives the user opportunities to adjust the image quality and the radiation exposure according to the needs for the actual examination. Automatic brightness control (ABC) is used to ensure that the brightness of the image at the monitor is constant. This is accomplished with automatic adjustment of tube voltage and current to accommodate the varying attenuation of the patient. There are at least two dose levels available and in most examinations adequate image quality is obtained using the low-dose mode [8, 9]. Low-dose technique is also applicable for cine-runs. It is advisable to always start fluoroscopy in low-dose mode and then switching to a higher dose level if necessary. In examinations of the peripheral parts of the patient, the ABC might not work satisfactorily and cause “image flare”. In these cases manual selection of exposure parameters or technique-lock of the ABC to a preferred setting is recommended. Use of technique-lock is also needed if radiation-opaque objects have to be inserted into the image field.

**Figure 1:**
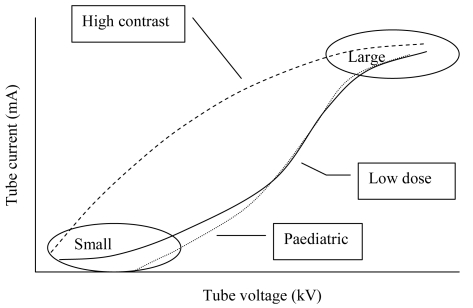
Different modes of operation for the regulation of tube voltage and tube current using automatic brightness control.

It is sometimes also possible to choose the mode of operation of the ABC. If low dose is a priority, the tube voltage is increased more than the current as the patient thickness increases. The increase in tube voltage will result in a slight decrease in image contrast especially for soft tissue. In situations where image contrast is crucial, the tube current could instead be increased more than the tube voltage (Figure 1). For paediatric use, it is desirable to have a low dose and therefore paediatric mode (if available) will provide a slightly higher tube voltage for thin patients. Theoretical studies [10] have shown that there is a potential for dose reduction in paediatric examinations by using a combination of low tube voltage and increased filtration (0.2 mm Cu). It is however difficult to accomplish this with present generators.

Proper algorithms for ABC function and use of the suitable mode is thus important both for patient dose (reduction of factor 2) and image quality [9]. Since it is not certain that these factors could be adjusted easily on the equipment, it is important to consider them during commissioning of the equipment.

**Figure 2:**
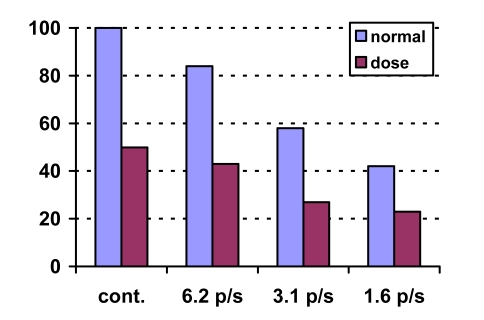
Example of dose levels for continuous and pulsed fluoroscopy.

A useful way of decreasing patient dose while maintaining image quality is to use pulsed fluoroscopy [9, 11], which produces radiation in short pulses, opposite to continuous mode. Pulse rates as low as single pulses per second can be chosen. Lower pulse rates will result in larger dose savings (Figure 2). A digital image memory and gap filling is used to obtain a continuous flicker-free video display on the monitor. The disadvantage of pulsed fluoroscopy is the loss of temporal resolution. With some training, this is however not a major problem.

Another method to reduce patient dose is to use frame averaging. In this case a series of frames produced by the detector are averaged before presentation on the monitor. This will reduce the noise in the presented image and therefore give the possibility to reduce the dose rate used without loss of image quality. A disadvantage using substantial frame averaging is the noticeable image lag.

Flat panel detectors have also been introduced for fluoroscopy. Characteristics of these detectors, such as high sensitivity to X-rays, large dynamic range and good contrast resolution, give the opportunity to optimize the examination technique with respect to absorbed dose and image quality. When introducing these systems, it is essential to explore the possibilities of reducing patient dose while maintaining adequate image quality, and not to improve image quality when it is not necessary [12]. It has been reported that the patient dose could be reduced by 30% using flat panel detectors. A prerequisite to capitalize on these possibilities is that the function of the equipment and the methodology is thoroughly reviewed [13].

## METHODOLOGY

Even though careful considerations on the functioning of the equipment will give the possibility to perform procedures with low patient doses, the main factor deciding the patient dose is the methodology used by the operator. Important factors in this respect are the fluoroscopy time, restriction of the radiation field and positioning of the patient.

Use of last-image-hold (LIH), which enables the last live image to be displayed continuously when the radiation is terminated, could reduce the fluoroscopy time to half compared to when it is not used. It enables the operator to examine the image as long as necessary without the use of radiation. Many types of equipment also have the possibility to see, on the LIH image, the effect of adjustment of the collimators on the image field. This further decreases the beam-on time. It has been shown that equally large dose savings can be obtained if appropriate restriction of the radiation field is employed [3, 14]. Reduction of a circular radiation field size from 20 cm to 16 cm will reduce the amount of radiation to the patient by about 40 percent.

Images needed for documentation should preferably be exposed using the fluoroscopy system since the absorbed dose needed is less compared to radiography.

For procedures employing cine runs it is equally important to limit the number of frames to what is essential for the examination. Short cine loops viewed repeatedly usually provide adequate information [3]. It is not uncommon for the length of the cine runs to increase when shifting from film to digital techniques [15]. This is probably because long runs no longer present a handling problem for the personnel.

There is a possibility to increase detail resolution by using magnification mode of the image intensifier. This will however decrease the brightness gain of the intensifier and the generator will compensate for this by increasing the exposure by the square of the magnification factor. Magnification mode should therefore not be used unless it is necessary to perform the procedure.

**Figure 3:**
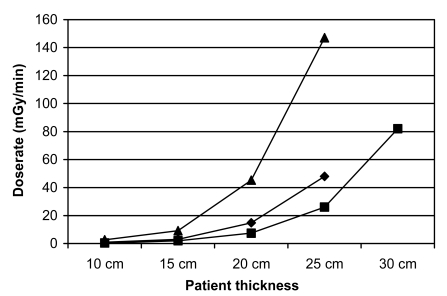
Entrance skin dose level dependent on patient thickness, tube voltage and focus-skin distance. For tube voltage 70 kV and focus-skin distance 70 cm (♦), 100kV, 70cm (■), 70 kV,40cm (▲). This figure gives an example. The actual dose rate depends on setting of the ABC.

Positioning of the patient with respect to the X-ray tube and the detector is very important not only for the possibility of visualising the anatomy but also for the image quality and to restrict the radiation dose to the patient. Tube angulations influence the exposure significantly due to the large effect on the projected path through the patient. Orientations giving rise to high dose rates should not be used more than absolutely necessary [3]. Integral to good practice is to position the patient as close as possible to the detector. ESD increases dramatically as the patient is moved towards the X-ray tube. If combined with thick patients, a short distance between X-ray tube and the patient will lead to very high dose rates (Figure 3). This could lead to infliction of radiation injury to the patient even with modest fluoroscopy time. In extensive interventional procedures it is advisable to reposition the equipment with respect to the patient during some occasions to avoid irradiation of the same part of the skin.

Transmission of the radiation through the patient can be increased if additional filtration of the beam is used. This has been applied for several types of examination [14, 16, 17] and substantial dose reductions (about 50%) have been reported. Usually, about 0.2 mm Cu is added to the original filtration of the radiation beam. Another way of reducing the patient dose is to remove the antiscatter grid. The grid not only removes scattered radiation but also a part of the primary radiation. The dose rate has to be increased by approximately a factor 2 when the grid is used. In small-sized patients such as small children, the amount of scattered radiation is also small and no grid is needed. It is therefore important that the grid is easily removable in equipments used for paediatric examinations. For medium-sized objects, an air gap could be used for scatter rejection instead of a grid. The reduction of radiation dose is also large for air gap technique but care has to be taken to avoid small distance between patient and X-ray tube [18]. For large-sized patients a grid is necessary to avoid deterioration of image contrast.

## RADIATION PROTECTION OF THE PERSONNEL

It is important to remember that the radiation dose to the personnel is directly related to the dose to the patient since the major contribution is scattered radiation from the patient. Intensity of the radiation is highest at the tube side of the patient and therefore the amount of scattered radiation is largest at this side. It is therefore advantageous to perform examinations with an undercouch tube whenever possible since this will reduce the amount of scattered radiation towards the head and chest. Staff working with fluoroscopy should have adequate radiation protection. This includes well designed aprons or vest and skirt, and thyroid protection if deemed necessary. Attenuation equivalent to 0.35 mm lead provides substantial protection even for those working with complex interventional procedures [19]. Light-weight aprons manufactured from non-lead materials provide adequate protection for those who do not have a heavy workload in the fluoroscopy room and will at the same time spare the spine and shoulders from the heavy weight of lead aprons. Ceiling mounted lead acrylic viewing screens will provide very good protection for the head and neck [20]. They are recommended for rooms where angiography and interventional work is performed.

For those performing interventional procedures it is very important to keep the hands out of the radiation field. This is especially so when working on the tube side of the patient.

## QUALITY SYSTEM

As discussed above, image quality and radiation dose are greatly influenced by technical and procedural factors. Image quality and dose are also linked and the optimisation of the procedures is not trivial. There should be a comprehensive quality system established involving physicians, staff and medical physicists to review both existing procedures and the introduction of new methods. A quality system should cover all aspects from procurement and quality control of the equipment, evaluation of methods and measurement of dose to patients and personnel to a program to ensure that everybody working with the radiological procedures have adequate knowledge on radiation protection and dose control techniques.
